# Supplementary material for: Plasma neurofilament light, glial fibrillary acid protein, and phosphorylated tau 181 as biomarkers for neuropsychiatric symptoms and related clinical disease progression
Source: Alzheimers Res Ther. 2024 Jul 25;16:165. doi: 10.1186/s13195-024-01526-4 (PMC11270946; doi:10.1186/s13195-024-01526-4)
Supplement: Supplementary file 1 — Supplementary Material 1 [file 13195_2024_1526_MOESM1_ESM.docx]

**Supplementary**

**Table S1**

**Title:** CSF core AD biomarkers in participants with and without NPS

**Description:** CSF Aβ_42_, tTau and pTau181 mean levels and mean ratios of pTau181/Aβ_42_ ± SD (standard deviation) are shown for participants with NPS compared to those without at baseline. Aβ_42_, beta-amyloid 1-42 peptide; tTau, total tau; pTau181, tau phosphorylated at threonine-181.

|  |  | **NPS +**  n=72 |  | **NPS -**  n=79 |  | **p** |
| --- | --- | --- | --- | --- | --- | --- |
| Aβ_42_ |  | 754.5 ± 278.2 |  | 979.6 ± 266.5 |  | < .001 |
| tTau |  | 537.5 ± 365.4 |  | 310.8 ± 206.4 |  | < .001 |
| pTau181 |  | 74.1 ± 33.3 |  | 55.9 ± 23.1 |  | < .001 |
| pTau181/ Aβ_42_ |  | .12 ± .10 |  | .06 ± .05 |  | < .001 |

**Table S2**

**Title:** Frequency of single items of the NPI-Q

**Description:** Frequency of the single items of the NPI-Q in participants with NPS. The number of participants are represented by n. NPS, neuropsychiatric symptoms; NPI-Q, neuropsychiatric inventory questionnaire

| **Participants with NPS** | **n=72 (%)** |
| --- | --- |
| Anxiety | 37 (51.4) |
| Apathy/Indifference | 30 (41.7) |
| Sleep and nighttime behavior disorders | 28 (38.9) |
| Irritability/Lability | 26 (36.1) |
| Depression/Dysphoria | 23 (31.9) |
| Appetite and eating disorders | 23 (31.9) |
| Agitation/Aggression | 18 (25.0) |
| Delusions | 9 (12.5) |
| Elation/Euphoria | 9 (12.5) |
| Disinhibition | 7 (9.7) |
| Aberrant motor behavior | 6 (8.3) |
| Hallucinations | 3 (4.2) |

**Table S3**

**Title:** Associations of plasma biomarkers with NPS severity and NPS severity change, considering cerebral AD pathology

**Description:** Results from the linear regression analysis showing the associations of plasma NfL, GFAP and pTau181 with NPS severity at baseline and follow-up (based on the NPI-Q total severity score) as well as the NPS severity change over time (defined through the ∆NPI-Q total severity score between baseline and follow-up/time to follow-up in months) after considering age, sex and AD pathology. A positive AD profile was defined based on a center cut-off of pTau181/Aβ42 ratio > 0.078. Beta coefficients, odds ratios, 95% confidence interval and p-values are shown. Aβ_42_, beta-amyloid 1-42 peptide; GFAP, glial fibrillary acid protein; NfL, neurofilament light chain; NPI-Q, neuropsychiatric inventory questionnaire; NPS, neuropsychiatric symptoms; pTau181, tau phosphorylated at threonine 181

|  | **baseline NPS** | | **future NPS** | | **NPS severity change** | |
| --- | --- | --- | --- | --- | --- | --- |
|  | **OR (95% CI)** | **p** | **OR (95% CI)** | **p** | **β (95% CI)** | **p** |
| NfL | n.s. | n.s. | n.s. | n.s. | 0.28 (0.05 - 0.51) | **.020*** |
| GFAP | n.s. | n.s. | 2.5 (1.3 – 5.0) | **.009*** | 0.37 (0.11 - 0.65) | **.006*** |
| pTau181 | n.s. | n.s. | n.s. | n.s. | n.s. | n.s. |

**Table S4**

**Title:** Associations of plasma biomarkers with NPS severity and NPS severity change stratified by cognitive status

**Description:** Results from the linear regression analysis showing the associations of plasma NfL, GFAP and pTau181 with NPS severity at baseline and follow-up (based on the NPI-Q total severity score) as well as the NPS severity change over time (defined through the ∆NPI-Q total severity score between baseline and follow-up/time to follow-up in months) after considering age and sex. Results are stratified by cognitive status ((A), cognitively impaired with CDR=0.5 vs. (B), cognitively unimpaired with CDR=0 at baseline). Beta coefficients, odds ratios, 95% confidence interval and p-values are shown. GFAP, glial fibrillary acid protein; NfL, neurofilament light chain; NPI-Q, neuropsychiatric inventory questionnaire; NPS, neuropsychiatric symptoms; pTau181, tau phosphorylated at threonine 181

|  | **baseline NPS severity** | | **future NPS severity** | | **NPS severity change** | |
| --- | --- | --- | --- | --- | --- | --- |
|  | **OR (95% CI)** | **p** | **OR (95% CI)** | **p** | **β (95% CI)** | **p** |
| NfL | 1.4 (0.8 – 2.5) | .240 | 1.2 (0.6 – 2.3) | .603 | 0.29 (-0.01 - 0.59) | .057 |
| GFAP | 1.2 (0.7– 2.1) | .576 | 4.5 (1.5 – 13.9) | **.007*** | 0.36 (0.03 - 0.71) | **.036*** |
| pTau181 | 1.3 (0.8 – 2.1) | .265 | 1.1 (0.6 – 1.9) | .760 | -0.07 (-0.36 - 0.22) | .630 |

**A Cognitively impaired participants**

**B Cognitively unimpaired participants**

|  | **baseline NPS severity** | | **future NPS severity** | | **NPS severity change** | |
| --- | --- | --- | --- | --- | --- | --- |
|  | **OR (95% CI)** | **p** | **OR (95% CI)** | **p** | **β (95% CI)** | **p** |
| NfL | 0.6 (0.3 – 1.2) | .147 | 2.1 (0.9 – 5.1) | .088 | 0.14 (-0.49 - 0.21) | .393 |
| GFAP | 0.8 (0.4 – 1.5) | .440 | 1.5 (0.6 – 3.6) | .402 | 0.18 (-0.15 – 0.52) | .269 |
| pTau181 | 1.0 (0.5 – 1.7) | .828 | 1.8 (0.7 – 4.5) | .976 | 0.26 (-0.05 - 0.57) | .099 |

**Figure S1**

**Title:** Correlation between plasma NfL, pTau181 and GFAP with NPS

**Description:** Scatter plot with regression line and 95% confidence interval showing the correlation between the plasma levels of NfL, pTau181 and GFAP with the NPS severity at baseline (A), follow-up (B) and the NPS severity change from baseline to follow-up visit (C). The NPI-Q total severity score (0-36) was used for (A) and (B) and the ∆NPI-Q total severity score/months to follow-up for (C). A negative score on the x-axis in (C) would mean an improvement, or less frequent NPS, while a positive and higher score indicate worsening and more severe and/or frequent NPS at follow-up. GFAP, glial fibrillary acid protein; NfL, neurofilament light chain; NPI-Q, neuropsychiatric inventory questionnaire; NPS, neuropsychiatric symptoms; pTau181, tau phosphorylated at threonine 181
