# Supplementary material for: Plasma neurofilament light, glial fibrillary acid protein, and phosphorylated tau 181 as biomarkers for neuropsychiatric symptoms and related clinical disease progression
Source: Alzheimers Res Ther. 2024 Jul 25;16:165. doi: 10.1186/s13195-024-01526-4 (PMC11270946; doi:10.1186/s13195-024-01526-4)
Supplement: Supplementary file 2 — Supplementary Material 2 [file 13195_2024_1526_MOESM2_ESM.docx]

**Supplementary**

**Table S1**

**Title:** CSF core AD biomarkers in participants with and without NPS

**Description:** CSF Aβ_42_, tTau and pTau181 mean levels and mean ratios of pTau181/Aβ_42_ ± SD (standard deviation) are shown for participants with NPS compared to those without at baseline. Aβ_42_, beta-amyloid 1-42 peptide; tTau, total tau; pTau181, tau phosphorylated at threonine-181.

|  |  | **NPS +**  n=72 |  | **NPS -**  n=79 |  | **p** |
| --- | --- | --- | --- | --- | --- | --- |
| Aβ_42_ |  | 754.5 ± 278.2 |  | 979.6 ± 266.5 |  | < .001 |
| tTau |  | 537.5 ± 365.4 |  | 310.8 ± 206.4 |  | < .001 |
| pTau181 |  | 74.1 ± 33.3 |  | 55.9 ± 23.1 |  | < .001 |
| pTau181/ Aβ_42_ |  | .12 ± .10 |  | .06 ± .05 |  | < .001 |

**Table S2**

**Title:** Frequency of single items of the NPI-Q

**Description:** Frequency of the single items of the NPI-Q in participants with NPS. The number of participants are represented by n. NPS, neuropsychiatric symptoms; NPI-Q, neuropsychiatric inventory questionnaire

| **Participants with NPS** | **n=72 (%)** |
| --- | --- |
| Anxiety | 37 (51.4) |
| Apathy/Indifference | 30 (41.7) |
| Sleep and nighttime behavior disorders | 28 (38.9) |
| Irritability/Lability | 26 (36.1) |
| Depression/Dysphoria | 23 (31.9) |
| Appetite and eating disorders | 23 (31.9) |
| Agitation/Aggression | 18 (25.0) |
| Delusions | 9 (12.5) |
| Elation/Euphoria | 9 (12.5) |
| Disinhibition | 7 (9.7) |
| Aberrant motor behavior | 6 (8.3) |
| Hallucinations | 3 (4.2) |

**Table S3**

**Title:** Associations of plasma biomarkers with NPS severity and NPS severity change, considering cerebral AD pathology

**Description:** Results from the linear regression analysis showing the associations of plasma NfL, GFAP and pTau181 with NPS severity at baseline and follow-up (based on the NPI-Q total severity score) as well as the NPS severity change over time (defined through the ∆NPI-Q total severity score between baseline and follow-up) after considering age, sex and AD pathology. A positive AD profile was defined based on a center cut-off of pTau181/Aβ42 ratio < 0.078. Beta coefficients, 95% confidence interval and p-values are shown. Aβ_42_, beta-amyloid 1-42 peptide; GFAP, glial fibrillary acid protein; NfL, neurofilament light chain; NPI-Q, neuropsychiatric inventory questionnaire; NPS, neuropsychiatric symptoms; pTau181, tau phosphorylated at threonine 181

|  | **baseline NPS severity** | | **future NPS severity** | | **NPS severity change** | |
| --- | --- | --- | --- | --- | --- | --- |
|  | **β (95% CI)** | **p** | **β (95% CI)** | **p** | **β (95% CI)** | **p** |
| NfL | n.s. | n.s. | 0.27 (0.06 – 0.48) | **.014*** | 0.24 (0.02 - 0.46) | **.035*** |
| GFAP | 0.18 (-0.03 – 0.33) | .052 | 0.25 (0.03 - 0.46) | **.025*** | 0.30 (0.08 - 0.52) | **.007*** |
| pTau181 | n.s. | n.s. | n.s. | n.s. | n.s. | n.s. |

**Table S4**

**Title:** Associations of plasma biomarkers with NPS severity and NPS severity change stratified by cognitive status

**Description:** Results from the linear regression analysis showing the associations of plasma NfL, GFAP and pTau181 with NPS severity at baseline and follow-up (based on the NPI-Q total severity score) as well as the NPS severity change over time (defined through the ∆NPI-Q total severity score between baseline and follow-up) after considering age and sex. Results are stratified by cognitive status (cognitively impaired with CDR=0.5 vs. cognitively unimpaired with CDR=0 at baseline). Beta coefficients, 95% confidence interval and p-values are shown. GFAP, glial fibrillary acid protein; NfL, neurofilament light chain; NPI-Q, neuropsychiatric inventory questionnaire; NPS, neuropsychiatric symptoms; pTau181, tau phosphorylated at threonine 181

|  | **baseline NPS severity** | | **future NPS severity** | | **NPS severity change** | |
| --- | --- | --- | --- | --- | --- | --- |
|  | **β (95% CI)** | **p** | **β (95% CI)** | **p** | **β (95% CI)** | **p** |
| NfL | -0.05 (-0.31 – 0.20) | .687 | 0.32 (0.34 – 0.61) | **.029*** | 0.30 (0.01 - 0.59) | **.044*** |
| GFAP | <-0.01 (<-0.01 – 0.47) | .052 | 0.30 (0.02 - 0.58) | **.039*** | 0.32 (0.04 - 0.60) | **.028*** |
| pTau181 | 0.10 (-0.14 – 0.34) | .401 | 0.20 (-0.08 - 0.48) | .623 | 0.05 (-0.23 - 0.34) | .707 |

**A Cognitively impaired participants**

**B Cognitively unimpaired participants**

|  | **baseline NPS severity** | | **future NPS severity** | | **NPS severity change** | |
| --- | --- | --- | --- | --- | --- | --- |
|  | **β (95% CI)** | **p** | **β (95% CI)** | **p** | **β (95% CI)** | **p** |
| NfL | 0.23 (-0.03 – 0.48) | .079 | 0.14 (-0.18 – 0.46) | .377 | 0.01 (-0.31 - 0.33) | .954 |
| GFAP | -0.12 (-0.37 – 0.13) | .336 | -0.02 (-0.32 – 0.28) | .887 | 0.07 (-0.23 – 0.37) | .631 |
| pTau181 | -0.10 (-0.34 – 0.15) | .427 | 0.10 (-0.19 - 0.40) | .485 | 0.20 (-0.09 - 0.49) | .178 |

**Figure S1**

**Title:** Correlation between plasma NfL, pTau181 and GFAP with NPS severity change over time

**Description:** Scatter plot with regression line and 95% confidence interval showing the correlation between the plasma levels of NfL, pTau181 and GFAP with the NPS severity change from baseline to follow-up visit, based on the ∆NPI-Q total severity score. The NPS severity change values are shown on the x-axis, whereas a negative score would mean an improvement, or less frequent NPS, while a positive and higher score indicate worsening and more severe and/or frequent NPS at follow-up. GFAP, glial fibrillary acid protein; NfL, neurofilament light chain; NPI-Q, neuropsychiatric inventory questionnaire; NPS, neuropsychiatric symptoms; pTau181, tau phosphorylated at threonine 181

*
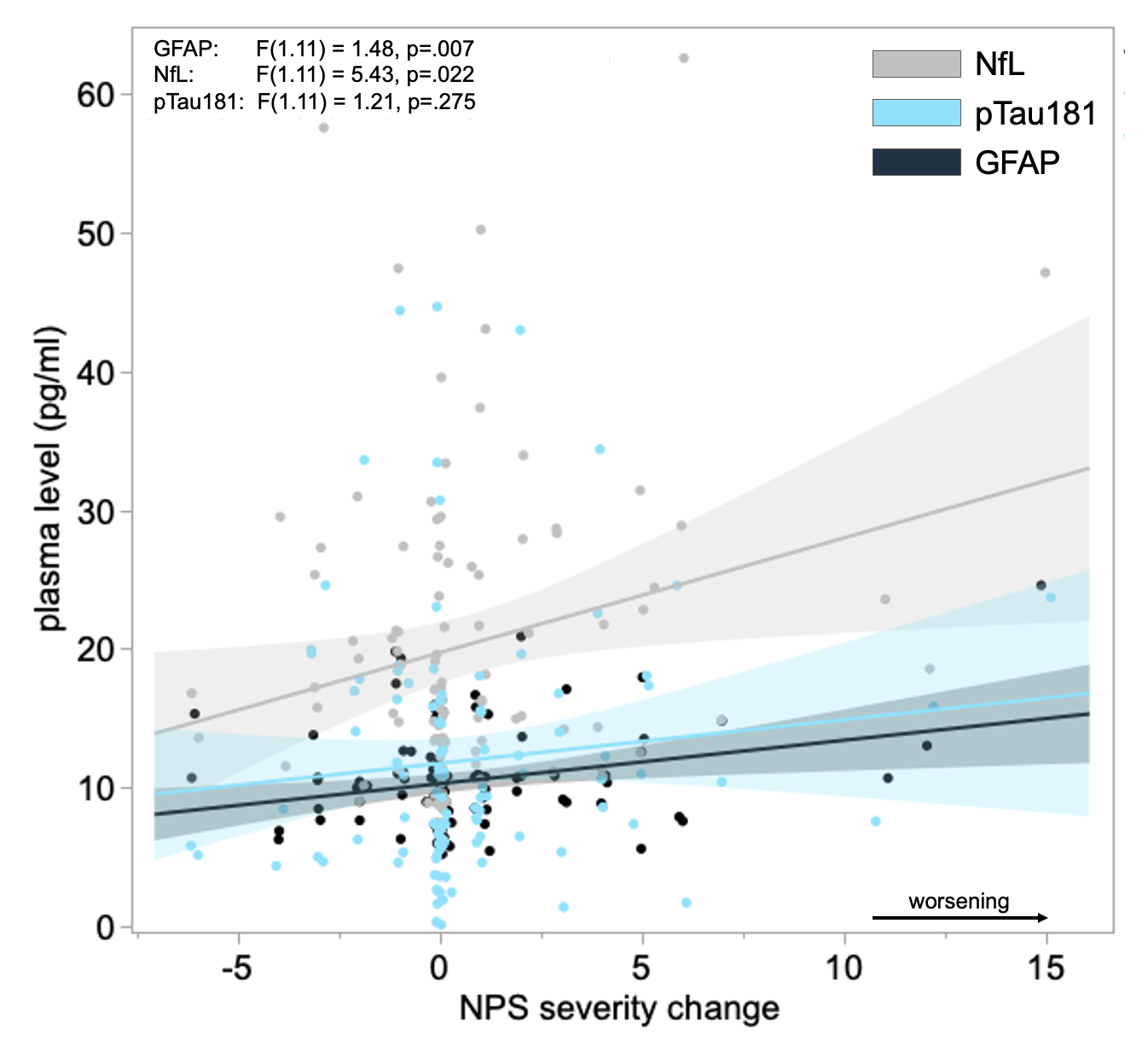
*
